# Severe acute respiratory syndrome coronavirus 2 (SARS-CoV-2) membrane (M) protein inhibits type I and III interferon production by targeting RIG-I/MDA-5 signaling

**DOI:** 10.1038/s41392-020-00438-7

**Published:** 2020-12-28

**Authors:** Yi Zheng, Meng-Wei Zhuang, Lulu Han, Jing Zhang, Mei-Ling Nan, Peng Zhan, Dongwei Kang, Xinyong Liu, Chengjiang Gao, Pei-Hui Wang

**Affiliations:** 1 Key Laboratory of Infection and Immunity of Shandong Province, Department of Immunology, School of Basic Medical Sciences, Cheeloo College of Medicine, Shandong University, 250012 Jinan, China; 2 Key Laboratory for Experimental Teratology of Ministry of Education and Advanced Medical Research Institute, Cheeloo College of Medicine, Shandong University, 250012 Jinan, China; 3 Department of Medicinal Chemistry, Key Laboratory of Chemical Biology (Ministry of Education), School of Pharmaceutical Sciences, Cheeloo College of Medicine, Shandong University, 44 West Culture Road, 250012 Jinan, Shandong PR China; 4 China–Belgium Collaborative Research Center for Innovative Antiviral Drugs of Shandong Province, 44 West Culture Road, 250012 Jinan, Shandong PR China; 5 Suzhou Research Institute, Shandong University, Shandong University, Suzhou, Jiangsu 215123 China

**Keywords:** Infectious diseases, Innate immunity

## Abstract

Coronavirus disease 2019 (COVID-19), caused by severe acute respiratory syndrome coronavirus 2 (SARS-CoV-2), has quickly spread worldwide and has affected more than 10 million individuals. A typical feature of COVID-19 is the suppression of type I and III interferon (IFN)-mediated antiviral immunity. However, the molecular mechanism by which SARS-CoV-2 evades antiviral immunity remains elusive. Here, we reported that the SARS-CoV-2 membrane (M) protein inhibits the production of type I and III IFNs induced by the cytosolic dsRNA-sensing pathway mediated by RIG-I/MDA-5–MAVS signaling. In addition, the SARS-CoV-2 M protein suppresses type I and III IFN induction stimulated by SeV infection or poly (I:C) transfection. Mechanistically, the SARS-CoV-2 M protein interacts with RIG-I, MAVS, and TBK1, thus preventing the formation of the multiprotein complex containing RIG-I, MAVS, TRAF3, and TBK1 and subsequently impeding the phosphorylation, nuclear translocation, and activation of IRF3. Consequently, ectopic expression of the SARS-CoV-2 M protein facilitates the replication of vesicular stomatitis virus. Taken together, these results indicate that the SARS-CoV-2 M protein antagonizes type I and III IFN production by targeting RIG-I/MDA-5 signaling, which subsequently attenuates antiviral immunity and enhances viral replication. This study provides insight into the interpretation of SARS-CoV-2-induced antiviral immune suppression and illuminates the pathogenic mechanism of COVID-19.

## Introduction

Coronavirus disease 2019 (COVID-19), caused by severe acute respiratory syndrome coronavirus 2 (SARS-CoV-2), has caused a vast number of infections and fatalities worldwide, constituting an acute and rapidly developing global health crisis. The sequence of SARS-CoV-2 has 79.5% identity with that of SARS-CoV-1 and ∼50% identity with that of Middle East respiratory syndrome coronavirus (MERS-CoV) at the whole-genome level.^1–3^ SARS-CoV-2, like SARS-CoV-1 and MERS-CoV, belongs to the betacoronavirus genus in the Coronaviridae family. Coronaviruses are single-stranded, positive-sense RNA viruses with the largest genomes (26–32 kb) among all RNA virus families and have a wide range of vertebrate hosts.^4^ Coronavirus transcripts have a 5′ cap structure and a 3′ poly(A) tail. SARS-CoV-2 is an enveloped virus with a genome of ∼30 kb. Upon entry of the virus into host cells, the viral genome is used as the template for replication, for transcription, and for the synthesis of positive-sense genomic RNA (gRNA) and subgenomic RNA (sgRNA). The gRNA is packaged into structures comprising the structural proteins, namely, the spike, membrane (M), and envelope proteins, to assemble progeny virions.^5^ Like SARS and MERS, COVID-19 may be life-threatening and typically begins with pneumonia.^3^ During the period 2002–2003, SARS-CoV-1 infected ∼8000 people, with an ∼11% fatality rate worldwide; since 2012, MERS-CoV has infected ∼2500 people with an ∼36% fatality rate.^6^ As of June 27, 2020, SARS-CoV-2 has infected 9,827,925 individuals and caused 494,841 deaths since its initial outbreak in December 2019, according to the COVID-19 Dashboard maintained by the Center for Systems Science and Engineering (CSSE) at Johns Hopkins University (https://coronavirus.jhu.edu/map.html). One of the hallmark clinical features of COVID-19 is poor protective immune response with high levels of proinflammatory cytokines, suggesting that the host immune system may be involved in COVID-19 pathogenesis.^7^

Innate immunity is the first line of host defense against viruses and is initiated by the recognition of pathogen-associated molecular patterns (PAMPs), such as single-stranded RNA (ssRNA), double-stranded RNA (dsRNA), and DNA, which trigger the production of type I interferons (IFN-α/β) and type III IFNs (/2/3) by infected cells.^8,9^ Toll-like receptor 3 (TLR3) senses dsRNA in endosomes, while retinoic acid-inducible gene I (RIG-I) and melanoma differentiation-associated gene 5 (MDA-5) are cytosolic receptors for dsRNA.^9^ Upon recognition of dsRNA, TIR-domain-containing adapter-inducing IFN-β (TRIF) is recruited to the cytoplasmic domain of TLR3. TRIF further associates with receptor-interacting protein 1 (RIP1), TNF receptor-associated factor 6 (TRAF6), and TANK-binding kinase 1 (TBK1). RIP1 and TRAF6 are involved in NF-κB pathway activation, whereas TBK1 directly phosphorylates the transcription factor IRF3, which is subsequently translocated to the nucleus, leading to the induction of genes encoding IFNs and other proinflammatory cytokines.^9,10^ When viruses enter host cells, viral dsRNA is recognized by RIG-I/MDA-5, which initiate an antiviral signaling cascade by interacting with mitochondrial antiviral signaling (MAVS, also called VISA/IPS-1/Cardif). MAVS then activates IκB kinase α/β (IKK) and TBK1/IKKε, which in turn activates the transcription factors NF-κB and IRF3, respectively, to induce the transcription of genes encoding IFNs and other proinflammatory cytokines.^11^ The cytosolic DNA sensor cyclic GMP–AMP (cGAMP) synthase (cGAS) can recognize dsDNA and produce 2′,3′-cGAMP, which can bind with stimulator of interferon genes (STING) and subsequently activate TBK1 and IRF3, leading to IFN production.^12^ Binding of type I or III IFNs to their specific receptors—type I IFN receptor (IFNAR) and type III IFN receptor (IFNLR), respectively—triggers the activation of receptor-associated Janus kinase 1 (JAK1)/tyrosine kinase 2 (TYK2), which stimulates the phosphorylation of STAT1 and STAT2.^9,13^ JAK2 also participates in type III IFN-induced STAT phosphorylation.^14^ Activated STAT1/STAT2 heterodimers associate with IRF9 to form the IFN-stimulated gene factor 3 (ISGF3) complex, which is in turn translocated into the nucleus and binds IFN-stimulated response elements (ISREs) in gene promoters, thus driving the expression of IFN-stimulated genes (ISGs) that endow host cells with antiviral abilities. Type I and III IFNs induce similar ISG signatures, although type I IFN signaling leads to more rapid induction of and decline in ISG expression.^9,13^

SARS-CoV-2 is a novel emerging coronavirus causing a global health threat. The means by which this virus is recognized by the innate immune system are currently unknown. However, studies of other coronaviruses have indicated that RIG-I/MDA-5 participate in sensing coronaviruses.^15^ In addition, TLR3 was shown to be involved in sensing SARS-CoV-1 in a mouse model.^8^ SARS-CoV-2 produces similar replication intermediates containing dsRNAs that can act as ligands for RIG-I/MDA5 or TLR3. Therefore, SARS-CoV-2 is likely detected by these dsRNA sensors, and this detection could be expected to induce the production of IFNs and other proinflammatory cytokines.^8^

Host antiviral immunity may induce selective pressure on viruses and has thus resulted in the distinct strategies used by viruses, including coronaviruses, to counteract IFN responses. Viral encoding of IFN antagonists is a common strategies for evading host antiviral immunity.^8^ SARS-CoV-1-encoded proteins, such as nonstructural protein 1, the papain-like protease domain in NSP3, ORF3b, ORF6, the M protein, and the nucleocapsid protein, have been suggested to antagonize IFNs and ISGs.^8^ In a recent screening study, SARS-CoV-2 M protein was shown to inhibit Sendai virus (SeV)-induced IFN-β promoter activation.^16^ The common respiratory virus influenza A virus also encodes an IFN antagonist, nonstructural protein 1, which blocks initial detection by PRRs through binding and masking aberrant RNAs produced during infection.^6^ The generation of IFN antagonists by viruses is a common strategy for evading host antiviral immunity.^8^ A striking clinical feature of COVID-19 is the drastic impairment of antiviral immunity.^6,17^ SARS-CoV-2 infection induces low-level expression of type I and III IFNs, accompanied by a moderate ISG response.^6^ In the severe cases of COVID-19, the expression of type I and III IFNs and ISGs, which exhibit immunopathogenic potential, are robustly triggered.^18,19^ It seems that the type I and III IFNs are suppressed in patients with moderate COVID-19, but still display elevated induction throughout the course of the disease in severe cases.^20^ Type I and III IFNs have been administered alone or in combination with other drugs to treat patients with COVID-19; this therapy has been shown to effectively suppress SARS-CoV-2 infection and may effectively prevent COVID-19.^21–24^ The replication of SARS-CoV-2 is dramatically reduced in Vero cells pretreated with recombinant IFN-α or IFN-β. Indeed, SARS-CoV-2 has been shown to be more sensitive than many other human pathogenic viruses, including SARS-CoV-1, to IFNs.^23,25^ Treatment with type I and III IFNs can dramatically inhibit SARS-CoV-2 replication in primary human airway epithelial cells, accompanied by the corresponding induction of ISGs.^26^ The role of type III IFNs in the suppression of viral replication has been emphasized.^27,28^ Type III IFN is a better therapeutic option than type I IFN for influenza A virus-induced disease due to its induction of antiviral immunity without proinflammatory responses.^29,30^ However, in some cases, type I and III IFN treatment promotes the replication of SARS-CoV-2; and phenotype is more pronounced in certain SARS-CoV-2 isolates.^31^ Two recent studies showed that the gene encoding ACE2, the cellular receptor for SARS-CoV-2, may be a novel ISG upregulated by type I IFNs to facilitate SARS-CoV-2 infection. ACE2 is indeed upregulated by IFN treatment and in COVID-19 patients.^32,33^ Therefore, the interactions between SARS-CoV-2 and the host type I and III IFN responses merit extensive investigation.

Overall, under most conditions, host type I and III IFNs play an important role in restricting SARS-CoV-2 infection and replication. For its defense, SARS-CoV-2 likely encodes multiple viral proteins to antagonize IFN responses, as do other coronaviruses. However, because of the recent emergence of SARS-CoV-2, information regarding the interaction between host antiviral immunity and SARS-CoV-2 infection is lacking. Thus, investigation of the mechanism by which SARS-CoV-2 evades the type I and III IFN responses is expected to contribute to the understanding of the pathogenesis and thus the treatment of COVID-19. Here, we reported that the SARS-CoV-2 M protein acts as an antagonist of both type I and III IFNs by affecting the formation of the RIG-I/MDA-5–MAVS–TRAF3–TBK1 signalosome, a multiprotein complex. The SARS-CoV-2 M protein inhibited type I and III IFN production induced by SeV infection, poly (I:C) transfection, and overexpression of RIG-I/MDA-5 pathway signaling molecules. Ectopic expression of the SARS-CoV-2 M protein facilitated the replication of vesicular stomatitis virus (VSV). This study revealed a previously unrecognized mechanism by which SARS-CoV-2 M protein evades host antiviral immunity, which may partially explain the clinical feature of impaired antiviral immunity in COVID-19 patients and provide insights into the pathogenicity and treatment of this virus.

## Results

### The SARS-CoV-2 M protein inhibits type I and III IFN induction by SeV and poly (I:C)

**Fig. 1:**
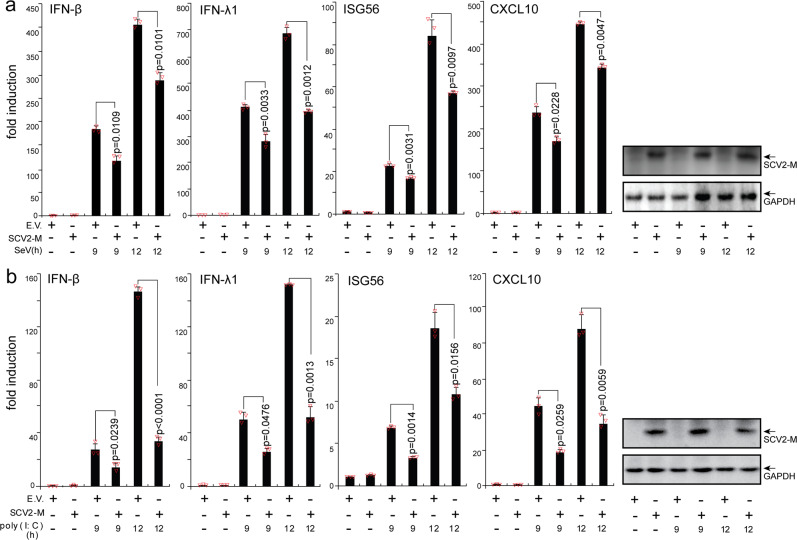
The SARS-CoV-2 M protein inhibits the induction of IFN-β, IFN-λ1, ISG56, and CXCL10 by SeV infection and poly (I:C) transfection. HEK293T cells cultured in 24-well plates (0.8–1 × 10^5^ cells per well) were transfected with pcDNA6B empty vector (E.V., 500 ng) or the SCV2-M plasmid (500 ng). Twenty-four hours after transfection, cells were stimulated by SeV infection (**a**) or poly (I:C) transfection (**b**) as indicated, and at 9 and 12 h after stimulation, the cells were harvested for RNA extraction and subsequent RT-qPCR analysis. Three independent biological replicates were analyzed, the results of one representative experiment are shown, and the error bars indicate the SD values. Statistical significance is shown as indicated. SARS-CoV-2 M protein SCV2-M; hours h

To explore whether the SARS-CoV-2 M protein affects type I and III IFN production, HEK293T cells expressing the SARS-CoV-2 M protein were infected with SeV or transfected with the dsRNA mimic poly (I:C). The expression levels of IFN-β, IFN-λ1, and two ISGs (IFN-stimulated 56 (ISG56) and CXCL10) were measured by RT-qPCR. Both SeV infection and poly (I:C) transfection strongly stimulated the expression of IFN-β, IFN-λ1, ISG56, and CXCL10 in control HEK293T cells. In HEK293T cells expressing the SARS-CoV-2 M protein, the induction of IFN-β, IFN-λ1, ISG56, and CXCL10 by SeV and poly (I:C) was significantly suppressed compared with that in HEK293T cells transfected with empty vector (Fig. 1). Moreover, SARS-CoV-2 M protein also inhibits poly (I:C)-induced production of IFN-β, IFN-λ1, ISG56, and CXCL10 in A549 cells (Supplemental Fig. S1a). Therefore, the SARS-CoV-2 M protein inhibits SeV- and poly (I:C)-induced upregulation of IFN-β, IFN-λ1, ISG56, and CXCL10, suggesting that it participates in antagonizing the IFN response.

### The SARS-CoV-2 M protein suppresses the cytosolic dsRNA-sensing pathway mediated by RIG-I/MDA-5 signaling

**Fig. 2:**
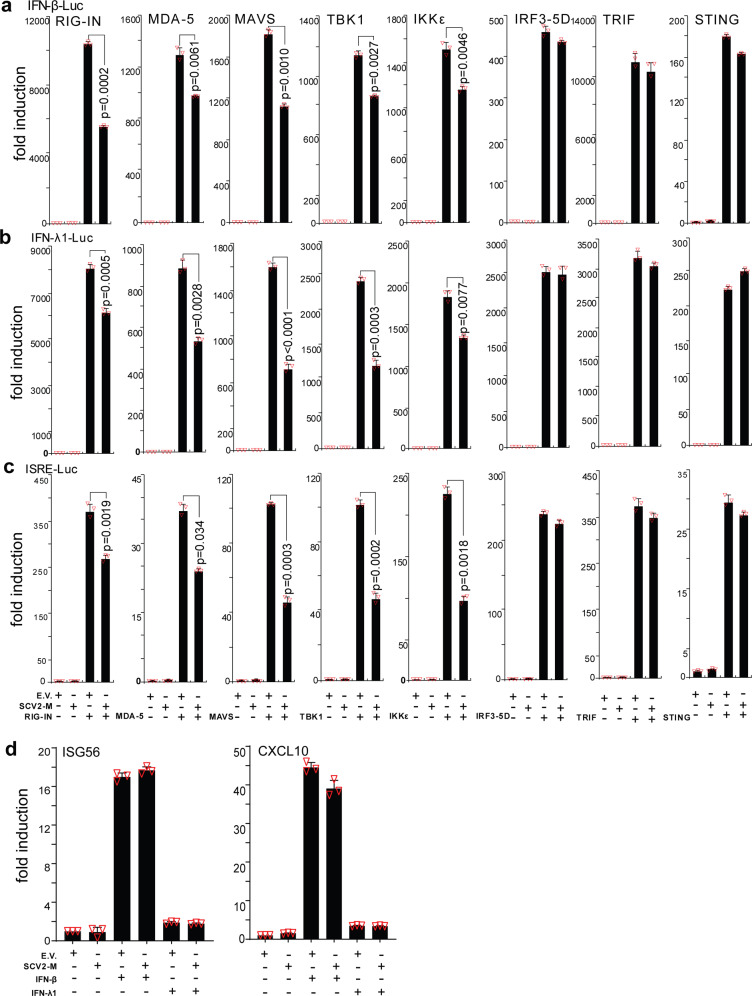
The SARS-CoV-2 M protein suppresses the activation of the luciferase reporters of type I and III IFNs and ISGs. The pcDNA6B empty vector and the SARS-CoV-2 M protein plasmids (100 ng) were transfected with the indicated combinations of plasmids expressing RIG-IN (100 ng), MDA-5 (100 ng), TBK1 (100 ng), IKKε (100 ng), IRF3-5D (100 ng, an active form of IRF3), TRIF (100 ng, component of TLR3-TRIF pathway), or STING (100 ng, component of cGAS-STING pathway) into HEK293T cells cultured in 48-well plates (0.5 × 10^5^ cells per well). The IFN-β-Luc (45 ng, the IFN-β luciferase reporter) (**a**), IFN-λ1-Luc (45 ng, the IFN-λ1 luciferase reporter) (**b**), or ISRE-Luc (45 ng, the IFN-stimulated response element luciferase reporter) (**c**) plasmids were also transfected to assess the activation of type I IFNs, type III IFNs, or ISGs, respectively. The pRL-TK (5 ng) was transfected into each well as an internal control. The pcDNA6B empty vector was used to normalize the total amount of transfected plasmid DNA. Dual‐luciferase assays were performed 36 h after transfection. **d** The pcDNA6B empty vector and the SARS-CoV-2 M protein plasmids (100 ng) were transfected into HEK293T cells as indicated. Twenty-four hours later, the cells were treated with recombinant human IFN-β (10 ng/mL) or IFN-λ1 (10 ng/mL). Three hours after simulation, the cells were harvested for RNA extraction and subsequent RT-qPCR analysis. Three independent biological replicates were analyzed, the results of one representative experiment are shown, and the error bars indicate the SD values. Statistical significance is shown as indicated. SARS-CoV-2 M protein SCV2-M

RIG-I and MDA-5 are cytosolic dsRNA sensors that participate in the recognition of SeV- and poly (I:C) and the subsequent induction of type I and III IFN production. To further confirm the inhibitory effect of the SARS-CoV-2 M protein on type I and III IFN expression, we employed luciferase reporters of type I and III IFN genes and ISGs. Specifically, we aimed to determine whether the SARS-CoV-2 M protein also interferes with activation of the cytosolic RNA-sensing pathway induced by overexpression of RIG-I/MDA-5 pathway components. The results of the IFN-β luciferase reporter (IFN-β-Luc) assays showed that overexpression of the SARS-CoV-2 M protein significantly suppressed the activity of IFN-β-Luc induced by RIG-IN (an active form of RIG-I), MDA-5, MAVS, TBK1, and IKKε but did not affect IRF3-5D (an active form of IRF3)-, TRIF-, or STING-induced IFN-β-Luc activation (Fig. 2a and Supplemental Fig. S2). Similarly, the SARS-CoV-2 M protein decreased the activity of IFN-λ1 luciferase reporter (IFN-λ1-Luc) and ISG luciferase reporter (ISRE-Luc) induced by RIG-IN, MDA-5, MAVS, TBK1, and IKKε but not by IRF3-5D, TRIF, or STING (Fig. 2b, c). Consistent with these results, in HEK293T cells expressing the SARS-CoV-2 M protein, ISG56 induction by RIG-I, MDA-5, MAVS, TBK1, and IKKε but not by IRF3-5D, TRIF, or STING was significantly suppressed (Supplemental Fig. S3). The inhibitory effect of SARS-CoV-2 M protein on the production of type I and III IFNs was also confirmed in A549 cells (Supplemental Fig. S1b). Taken together, these results indicate that the SARS-CoV-2 M protein can inhibit activation of the RIG-I/MDA-5–dependent cytosolic dsRNA-sensing pathway but does not affect the TLR3-TRIF signaling-mediated endosomal dsRNA-sensing pathway or the cGAS-STING signaling-mediated cytosolic DNA-sensing pathway. Because TBK1 is also downstream of TLR3-TRIF and cGAS-STING signaling, the SARS-CoV-2 M protein might inhibit dsRNA-induced IFN production at the step or upstream of TBK1.

### Subcellular localization of the SARS-CoV-2 M protein

**Fig. 3:**
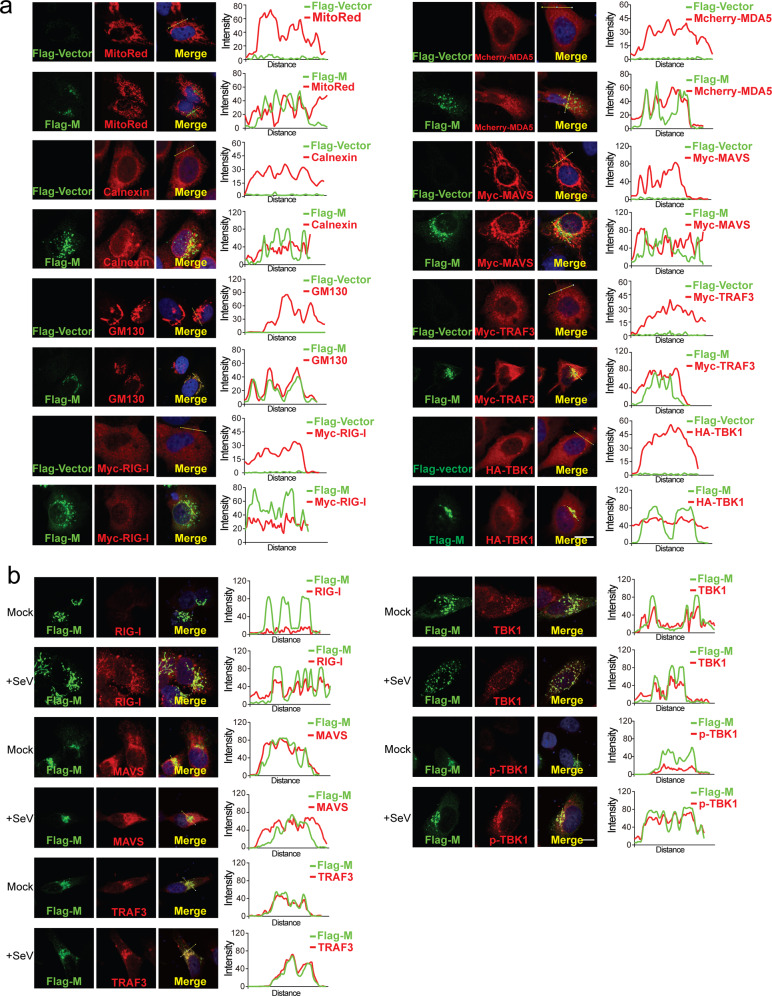
Subcellular localization of the SARS-CoV-2 M protein. HeLa cells seeded on coverslips in 12-well plates were transfected with the indicated plasmids. Twenty hours later, the cells were fixed, blocked, and then incubated with a rabbit anti-Flag antibody and a mouse antibody against the corresponding organelle marker or the indicated protein (**a**). HeLa cells in (**b**) were infected with SeV as indicated for 8 h before fixation, blocking and antibody incubation. Subsequently, the proteins were stained with a fluorescence-labeled secondary antibody. MDA-5 was visualized with a mCherry tag. (Right) Intensity profiles of SARS-CoV-2 M protein (Flag-M) and indicated proteins along the plotted lines, as analyzed by Image J line scan analysis. Nucleus were visualized with DAPI (blue). Confocal imaging results are representative of two independent experiments. Scale bar, 10 μm. MitoRed, mitochondria marker; Calnexin, ER marker; CM130, Golgi marker

The SARS-CoV-2 M protein is predicted to contain three transmembrane motifs at the N terminus (Supplemental Fig. S4). Therefore, we sought to determine the subcellular localization of the SARS-CoV-2 M protein. To this end, the Flag-tagged SARS-CoV-2 M protein was overexpressed in HeLa cells, reacted with the anti-Flag antibody and stained with a fluorescently labeled secondary antibody. The subcellular localization of the SARS-CoV-2 M protein was assessed by confocal microscopy. The SARS-CoV-2 M protein exhibited almost no colocalization with mitochondrial markers but was primarily localized to the endoplasmic reticulum (ER) and Golgi (Fig. 3a). Mitochondria, the ER, and the Golgi are important platforms for multiprotein complex formation and RIG-I/MDA-5 pathway signal transduction; therefore, we assessed the colocalization of the SARS-CoV-2 M protein with overexpressed RIG-I, MDA-5, MAVS, TRAF3, and TBK1 (Fig. 3b). Considering that overexpressed proteins might affect their natural distributions in the cells, we also used the protein-specific antibodies to explore the colocalization of SARS-CoV-2 M with these proteins in resting and SeV-infected HeLa cells (Fig. 3c). The signal intensities showed that SARS-CoV-2 M was strongly colocalized with TBK1 and TRAF3 (Fig. 3b) but only partially colocalized with RIG-I, MDA-5, and MAVS (Fig. 3a and b).

### The SARS-CoV-2 M protein interacts with RIG-I, MDA-5, MAVS, and TBK1

**Fig. 4:**
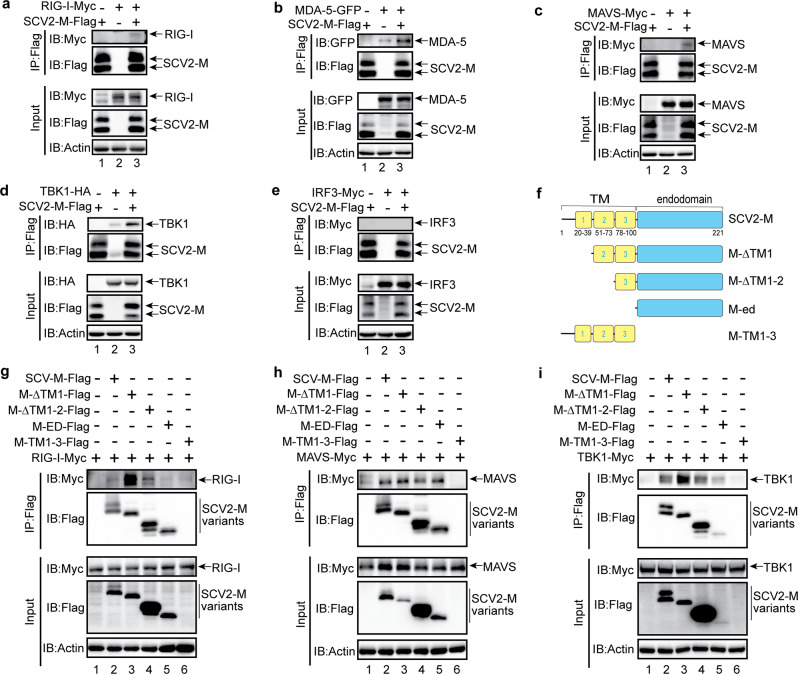
The SARS-CoV-2 M protein interacts with RIG-I (**a**), MDA-5 (**b**), MAVS (**c**), and TBK1 (**d**) but not with IRF3 (**e**). **f** The truncated variants of SARS-CoV-2 M protein are depicted. **g–i** The interaction of RIG-I, MAVS, and TBK1 with the truncated variants of SARS-CoV-2 M protein. The HEK293T cells were transfected with the indicated plasmids for 24 h before coimmunoprecipitation with the anti-Flag magnetic beads. The pcDNA6B empty vector was used to balance the total amount of plasmid DNA in the transfection. The input and immunoprecipitates were immunoblotted with the indicated antibodies. Immunoblotting results are representative of two independent experiments. SARS-CoV-2 M protein, SCV2-M

To identify the mechanism by which the SARS-CoV-2 M protein affects IFN signaling activation, coimmunoprecipitation (Co-IP) experiments were performed to assess interactions between SARS-CoV-2 M and RLR signaling molecules. We observed colocalization of the SARS-CoV-2 M protein with MAVS and TBK1; thus, we examined the interactions between the SARS-CoV-2 M protein and RIG-I, MDA-5, MAVS, and TBK1. In HEK293T cells, the SARS-CoV-2 M protein coexpressed with RIG-I, MDA-5, MAVS, or TBK1 (Fig. 4a–e), and Co-IP experiments were performed. RIG-I, MDA-5, MAVS, and TBK1 but not IRF3 were detected in SARS-CoV-2 M protein immunoprecipitates (Fig. 4a–e), indicating that the SARS-CoV-2 M protein can interact with RIG-I, MDA-5, MAVS, and TBK1 but not IRF3. Next, we generated the truncated variants of SARS-CoV-2 M protein (Fig. 4f) and investigated which region is responsible to the binding with RIG-I, MAVS, and TBK1. The results indicate that the endodomain of SARS-CoV-2 M protein lacking the TM (Fig. 4g, lane 5) could not bind with RIG-I, as a contrast, all the truncated variants of SARS-CoV-2 M protein can still interact with MAVS (Fig. 4h) and TBK1 (Fig. 4i); thus, the TM domain of SARS-CoV-2 M protein plays a pivotal role in interacting with RIG-I. Consistent with the Co-IP results, we observed strong colocalization of SARS-CoV-2 M with TBK1, partial colocalization of SARS-CoV-2 M with MAVS, and no colocalization of SARS-CoV-2 M with IRF3 (Figs. 3b and 6b).

### The SARS-CoV-2 M protein prevents the RIG-I–MAVS, MAVS–TBK1, and TRAF3–TBK1 interactions and inhibits IRF3 phosphorylation

We observed the interactions between the SARS-CoV-2 M protein and RIG-I, MDA-5, MAVS, and TBK1 (Fig. 4a–d). However, we could not detect the interaction between SARS-CoV-2 M and IRF3 (Fig. 4e). IRF3 activation and subsequent IFN production rely on the assembly of a multiprotein complex containing the dsRNA sensors RIG-I/MDA-5, MAVS, TRAF3, and TBK1. Because the SARS-CoV-2 M protein can interact with RIG-I, MDA-5, MAVS, and TBK1, we sought to investigate whether it affects RIG-I/MDA-5–MAVS–TRAF3–TBK1 complex formation, which is essential for IRF3 activation and IFN induction.

**Fig. 5:**
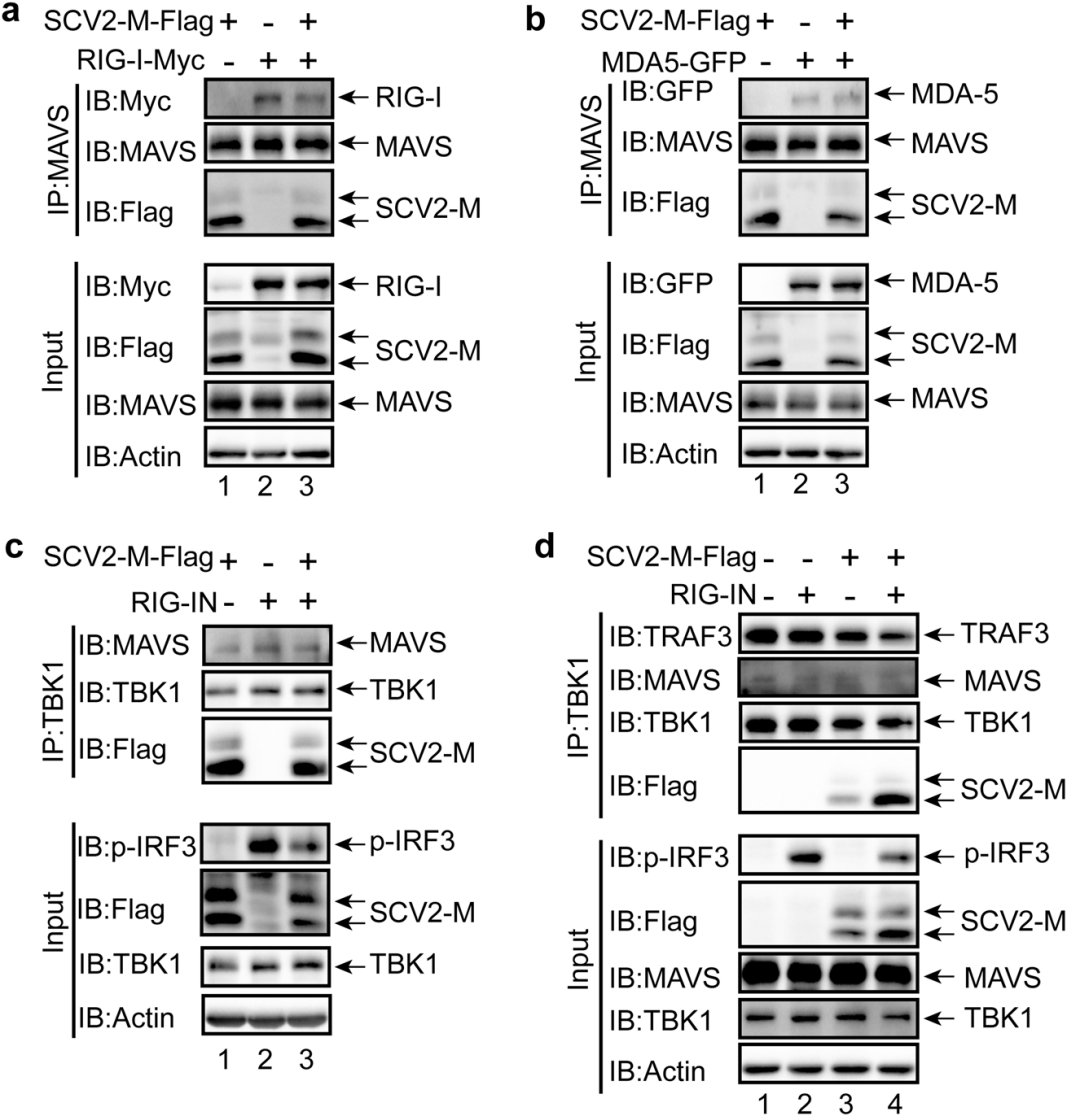
The SARS-CoV-2 M protein prevents the formation of the RIG-I–MAVS–TRAF3–TBK1 multiprotein complex. The SARS-CoV-2 M protein inhibits the RIG-I–MAVS (**a**), MAVS–TBK1 (**c**), and TRAF3–TBK1 (**d**) but not MDA-5–MAVS (**b**) interactions. The HEK293T cells were transfected with the indicated plasmids for 24 h before coimmunoprecipitation with the antibodies against MAVS (**a** and **b**) or TBK1 (**c** and **d**). The pcDNA6B empty vector was used to balance the total amount of plasmid DNA in the transfection. The input and immunoprecipitates were immunoblotted with the indicated antibodies. Immunoblotting results are representative of two independent experiments. SARS-CoV-2 M protein, SCV2-M

The SARS-CoV-2 M protein plasmid and plasmids expressing RIG-I or MDA-5 were cotransfected into HEK293T cell, 24 h later, MAVS antibodies were used to perform Co-IP. When the SARS-CoV-2 M protein was overexpressed, the binding between RIG-I and MAVS was reduced (Fig. 5a, lanes 2 compared to lane 3); however, in the same condition, the interaction between MDA-5 and MAVS was not affected (Fig. 5b, lanes 2 compared to lane 3), indicating that the SARS-CoV-2 M protein impedes the complex formation of RIG-I and MAVS but has no effect on the interaction between MDA-5 and MAVS.

In the endogenous Co-IP assay with the anti-TBK1 antibody, MAVS was detected in the immunoprecipitate (Fig. 5c and d). When the SARS-CoV-2 M protein was overexpressed in HEK293T cells, the amount of TBK1-associated MAVS was apparently reduced compared with that in cells not expressing the SARS-CoV-2 M protein (Fig. 5c, lanes 1 and 3 compared to lane 2; Fig. 5d, lane 4 compared to lane 2), suggesting that the SARS-CoV-2 M protein might preferentially inhibit the formation of the MAVS–TBK1 complex. Notably, when the formation of the TBK1–MAVS protein complex was reduced, IRF3 phosphorylation induced by RIG-IN was correspondingly impaired (Fig. 5c, lane 3 compared to lane 2). Similarly, the endogenous Co-IP assay with the anti-TBK1 antibody indicated that overexpression of the SARS-CoV-2 M protein impeded the endogenous association between TRAF3 and TBK1 (Fig. 5d, lane 4 compared to lane 2), thus correspondingly suppressing IRF3 phosphorylation (Fig. 5d, lane 4 compared to lane 2).

Previous studies have shown that the SARS-CoV-1 M protein and MERS-CoV M protein can suppress the interaction between TBK1 and TRAF3 to interfere with IFN production.^34,35^ Here, we found that the overexpression of the SARS-CoV-2 M protein not only suppressed the interaction between TBK1 and TRAF3 but also reduced the binding between RIG-I and MAVS (Fig. 5a) and the binding between MAVS and TBK1 (Fig. 5c and d), suggesting that the SARS-CoV-2 M protein affects the formation of the RIG-I–MAVS–TRAF3–TBK1 multiprotein complex and subsequent phosphorylation or IRF3.

### The SARS-CoV-2 M protein suppresses SeV-induced IRF3 phosphorylation and nuclear translocation

**Fig. 6:**
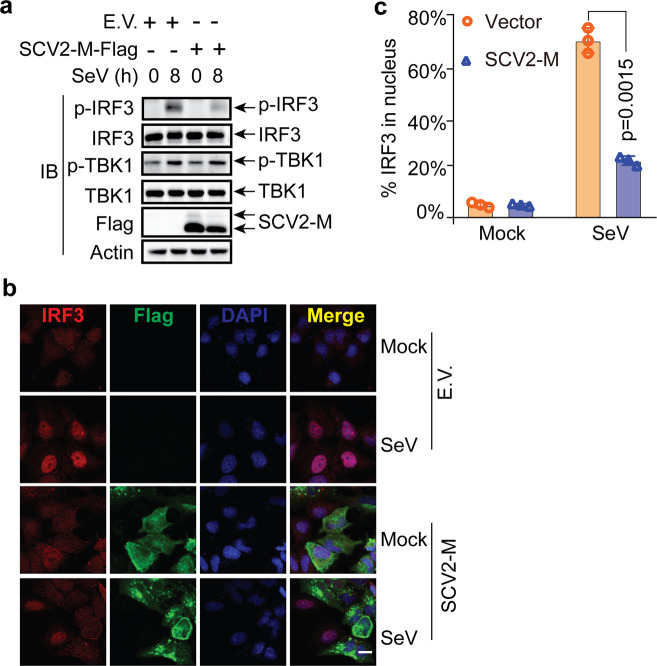
The SARS-CoV-2 M protein suppresses the phosphorylation and nuclear translocation of IRF3. **a** The SARS-CoV-2 M protein affects the phosphorylation of IRF3 upon SeV infection. HeLa cells seeded in six-well plates were transfected with the empty vector or Flag-tagged SARS-CoV-2 M protein plasmids for 20 h before infection with SeV (50 HA/mL). At the indicated time points, the cells were harvested and processed for immunoblotting with the indicated antibodies. **b** The SARS-CoV-2 M protein prevents the nuclear translocation of IRF3. HeLa cells seeded on coverslips in 12-well plates were transfected with the Flag empty vector or Flag-tagged SARS-CoV-2 M protein plasmids for 20 h before infection with SeV. After infection for 8 h, the coverslips were removed and processed for immunofluorescence staining with the mouse anti-Flag antibody and a rabbit anti-IRF3 antibody. Scale bar, 10 μm. **c** Quantification of the percentage of IRF3 in the nucleus upon SeV infection. IRF3 molecules in the nuclei in 50 cells per group were counted, and percentages were calculated. Experiments were performed in triplicate and calculated as the means ± SD. Immunoblotting (**a**) and confocal imaging (**b**) results are representative of two independent experiments. Empty vector EV, SARS-CoV-2 M protein SCV2-M, hours h

The phosphorylation and nuclear translocation of IRF3 is the hallmark of its activation, which is essential for type I and III IFN induction during viral infection. IRF3 phosphorylation is a prerequisite for both its nuclear translocation and activation of IFN transcription. Therefore, we next investigated the effect of the SARS-CoV-2 M protein on IRF3 phosphorylation. The results of RT-qPCR analysis (Fig. 1) and a luciferase reporter assays (Fig. 2) indicated that the SARS-CoV-2 M protein can inhibit the induction of type I and III IFN genes and that overexpression of the SARS-CoV-2 M protein reduces RIG-IN-induced IRF3 phosphorylation (Fig. 5c and d). However, whether the SARS-CoV-2 M protein affects IRF3 phosphorylation in an in vivo viral infection is unknown, but this knowledge may contribute to our understanding of the role of the SARS-CoV-2 M protein in SARS-CoV-2 infection. Because we did not have access to a biosafety level 3 laboratory, we performed viral infection studies with another RNA virus, SeV, as a proxy for SARS-CoV-2. To address the effect of the SARS-CoV-2 M protein on virus-induced IRF3 phosphorylation, HeLa cells and HeLa cells expressing the SARS-CoV-2 M protein were infected with SeV. The immunoblot results indicated that SeV infection can induce the phosphorylation of IRF3 in HeLa cells, although IRF3 phosphorylation was clearly decreased in HeLa cells expressing the SARS-CoV-2 M protein (Fig. 6a). Therefore, the SARS-CoV-2 M protein can inhibit IRF3 phosphorylation induced by SeV infection. In contrast, TBK1 phosphorylation was not affected in HeLa cells expressing the SARS-CoV-2 M protein (Fig. 6a).

The phosphorylation of IRF3 is a pivotal step required for its nuclear translocation to activate the transcription of type I and III IFNs. Because we observed that the SARS-CoV-2 M protein suppressed SeV-induced IRF3 phosphorylation, we next examined the effect of the SARS-CoV-2 M protein on SeV-induced IRF3 nuclear translocation. In uninfected HeLa cells, IRF3 was evenly distributed throughout the cytosol in either the absence or presence of the SARS-CoV-2 M protein (Fig. 6b), and SeV infection strongly stimulated the nuclear translocation of IRF3 in HeLa cells transfected with the control empty vector (Fig. 6b). However, SeV-induced IRF3 nuclear translocation was significantly decreased in HeLa cells expressing the SARS-CoV-2 M protein compared with the corresponding control cells. (Fig. 6b and c). Thus, the SARS-CoV-2 M protein exerts its inhibitory effect on type I and III IFNs by preventing IRF3 phosphorylation and nuclear translocation.

### The SARS-CoV-2 M protein promotes viral replication

**Fig. 7:**
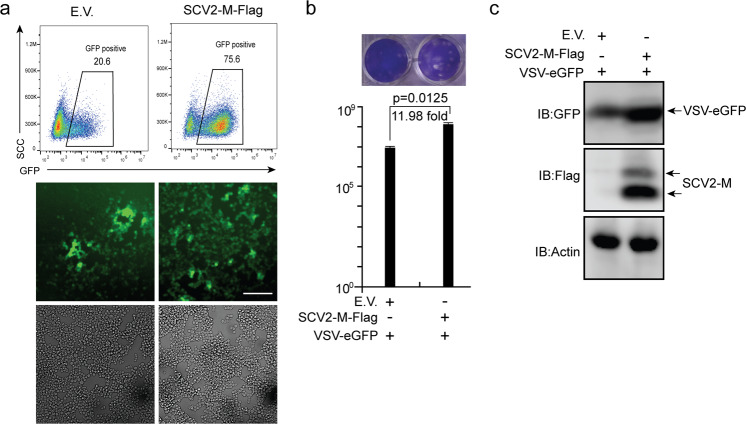
The SARS-CoV-2 M protein facilitates viral replication. The HEK293T cells were transfected with plasmids as indicated. Twenty-four hours later, the cells were infected with VSV-eGFP (MOI = 0.001). Twelve hours after infection, GFP-positive cells were visualized and analyzed with flow cytometry (**a**), and the culture supernatant (20 h post-infection) was collected for plaque assays to measure the titer of extracellular VSV-eGFP (PFU/mL) (**b**). Confocal imaging and flow cytometry results are representative of two independent experiments. Scale bar, 50 μm. Three independent biological replicates were analyzed (**a**); the results of one representative experiment are shown, and the error bars indicate the SD value. The statistical significance is shown as indicated. **c** The replication of intracellular VSV-eGFP in the cell lysate (20 h post-infection) was determined by immunoblotting using an anti-GFP antibody. Empty vector EV; SARS-CoV-2 M protein SCV2-M

We showed that the SARS-CoV-2 M protein can suppress type I and III IFN-induced antiviral immunity. Next, we sought to determine the role of the SARS-CoV-2 M protein in viral replication. VSV is commonly used as a model virus to study the effect of IFNs on viral replication. Here, HEK293T cells transfected with empty vector or the SARS-CoV-2 M protein expression plasmid were infected with VSV-eGFP. Via flow cytometry and fluorescence microscopy, we observed greater numbers of VSV-eGFP-positive cells in SARS-CoV-2 M protein-expressing cells than in control cells transfected with empty vector (Fig. 7a). In the culture supernatant of HEK293T cells expressing the SARS-CoV-2 M protein, the VSV-eGFP titer was much higher than that in the supernatant of HEK293T cells transfected with empty vector (Fig. 7b). Moreover, eGFP protein expression was higher in HEK293T cells expressing the SARS-CoV-2 M protein than in HEK293T cells transfected with empty vector (Fig. 7c). Thus, overexpression of the SARS-CoV-2 M protein facilitates the replication of VSV-eGFP.

## Discussion

Our previous studies have shown that innate antiviral immunity may play an important role in the SARS-CoV-2 clearance observed in COVID-19.^36^ SARS-CoV-2–mediated dysregulation of innate antiviral immunity and inflammatory responses is largely responsible for human deaths caused by COVID-19.^6,18^ Type I and III IFNs are typically suppressed in COVID-19 patients; however, the molecular mechanism of this phenomenon induced by SARS-CoV-2 needs to be elucidated.^6,27^ Here, we reported that the SARS-CoV-2 M protein can target the cytosolic RNA-sensing pathway of RIG-I/MDA-5 signaling to block the activation of type I and III IFN responses, thus inhibiting host antiviral immunity.

The M proteins of SARS-CoV-1 and MERS-CoV have been reported to suppress type I IFN production by interacting with TRAF3 to disrupt the TRAF3–TBK1 association.^34,35,37^ However, the MERS-CoV M protein exhibits extremely low IFN-antagonizing activity. The SARS-CoV-1 M protein interacts with TBK1, but no interaction between the MERS-CoV M protein and TBK1 is detectable, suggesting that these two highly pathogenic coronaviruses have begun to engage in distinct mechanisms of IFN suppression. Moreover, the M protein of the human coronavirus HKU1 does not impact type I IFN production, indicating that the IFN-antagonistic property of M proteins is not evolutionarily conserved across human coronaviruses.^34,35,37^ Importantly, one recent study found that the SARS-CoV-2 M protein cannot inhibit the production of type I IFN and ISG protein products.^38^ The differences between the M proteins of these coronaviruses in antagonizing IFN signaling prompted us to study the function and mechanism by which the SARS-CoV-2 M protein facilitates the evasion of innate antiviral immunity. We first examined the effect of the SARS-CoV-2 M protein on the activation of the RIG-I/MDA-5–MAVS signaling-mediated cytosolic dsRNA-sensing pathway and the TLR3–TRIF signaling-mediated endosomal dsRNA-sensing pathway. The results demonstrated that the SARS-CoV-2 M protein significantly inhibited the activation of type I and III IFN production induced by RIG-I/MDA-5 signaling components, including RIG-I, MDA-5, MAVS, TBK1, and IKKε, but not that induced by TRIF, the adaptor protein in TLR3–TRIF signaling, or STING, the adaptor protein in cGAS–STING signaling (Fig. 2). These results indicated that the SARS-CoV-2 M protein specifically targets the RIG-I/MDA-5-dependent RNA-sensing pathway but not the TLR3–TRIF-dependent endosomal dsRNA-sensing pathway or the cGAS–STING-dependent cytosolic dsDNA-sensing pathway. TLR3 is the major sensor of viral ligands in the lung to induce IFN production.^19^ However, SARS-CoV-2 M protein does not have any effect on TLR3–TRIF signaling activated IFN production; thus, there might be other SARS-CoV-2 proteins such as ORF9b are responsible to the inhibition of IFNs in the lung.^39^ Furthermore, Co-IP assays showed that the SARS-CoV-2 M protein interacts with RIG-I, MDA-5, MAVS, and TBK1 but not with IRF3 (Fig. 4). Overexpression of the SARS-CoV-2 M protein prevented the formation of the RIG-I–MAVS, MAVS–TBK1, and TRAF3–TBK1 complexes (Fig. 5). Thus, the SARS-CoV-2 M protein exerts its inhibitory effect on IFN production by impeding the formation of protein complexes containing RIG-I, MAVS, TRAF3, and TBK1, which are essential for IRF3 activation and IFN production. Moreover, we found that the SARS-CoV-2 M protein can inhibit the phosphorylation of IRF3 but not TBK1 (Fig. 6a), which may explain why it interacts with TBK1 but cannot suppress TRIF- or STING-induced IFN production.

Although the SARS-CoV-1 and MERS-CoV M proteins were shown to inhibit IRF3 phosphorylation, whether they also affect the nuclear translocation of IRF3 is unknown.^34,35,37^ We sought to extend the understanding of the biological function of the SARS-CoV-2 M protein and found that after SeV infection, IRF3 is mainly retained in the cytosol in HeLa cells expressing the SARS-CoV-2 M protein; in contrast, in control HeLa cells that did not express the SARS-CoV-2 M protein, IRF3 was predominantly translocated into the nucleus upon SeV infection (Fig. 6b and c). Thus, the SARS-CoV-2 M protein can potently inhibit the nuclear localization of IRF3 induced by SeV. Consistent with this finding, IRF3 phosphorylation in HeLa cells expressing the SARS-CoV-2 M protein was also attenuated compared with that in cells expressing empty vector (Fig. 6a).

The SARS-CoV-2 M protein contains three transmembrane motifs (Supplementary Fig. 4). We showed that this protein can colocalize with markers of the ER and Golgi apparatus (Fig. 3a), which are important signaling platforms in innate antiviral immunity.^40,41^ In addition, we observed that the SARS-CoV-2 M protein was colocalized with TBK1, partially colocalized with MAVS, but not colocalized with IRF3 (Figs. 3b and 6b); these observations were consistent with the results of our Co-IP assays indicating that the SARS-CoV-2 M protein interacts with RIG-I, MAVS, and TBK1 but not with IRF3 (Fig. 4). Moreover, the SARS-CoV-2 M protein impeded the formation of the RIG-I–MAVS–TBK1 multiprotein complex (Fig. 5a–d). An interesting question is whether this inhibition results from the suppression of cellular compartment translocation. A previous study suggested that the translocation of TBK1 from the ER or mitochondria to Golgi fragments is pivotal for SeV-induced IFN activation.^41^ Since the SARS-CoV-2 M protein is localized in both the ER and Golgi, its inhibitory effect is likely achieved by suppression of TBK1 translocation from the ER to Golgi compartments and subsequently affecting the formation of TBK1-containing Golgi fragments.^40,41^ Therefore, whether the formation of TBK1-containing Golgi fragments is affected by the SARS-CoV-2 M protein, which may constitute a novel mechanism by which virus-encoded proteins render IFN production inefficient, warrants further analysis.

The M proteins of SARS-CoV-1 and MERS-CoV have been reported to inhibit type I IFN production.^34,35,37^ However, the roles of coronavirus M proteins in viral infection and replication need further investigation. The results of flow cytometry, fluorescence microscopy, plaque assays, and immunoblotting indicated that the replication of VSV-eGFP was significantly enhanced in HEK293T cells expressing the SARS-CoV-2 M protein (Fig. 7). Thus, the SARS-CoV-2 M protein likely promotes SARS-CoV-2 replication by suppressing the host IFN response.

Relative to previous studies on the function of coronavirus M proteins in IFN antagonism, we provided several novel findings. First, we provided the first demonstration that the SARS-CoV-2 M protein suppresses the production of both type I IFN and type III IFN; the previous studies focused only on the effect of coronavirus M proteins on type I IFN. This finding may explain the advantage of type III IFNs in curing COVID-19.^28^ Second, the SARS-CoV-1 and MERS-CoV M proteins have been reported to impede TRAF3–TBK1 complex. In our study, we found that the SARS-CoV-2 M protein not only prevents TRAF3–TBK1 complex formation but also impairs RIG-I–MAVS–TBK1 multiprotein complex formation, which is essential for IRF3 phosphorylation, IRF3 translocation, and IFN transcription. Third, we observed that the SARS-CoV-2 M protein specifically inhibits RIG-I/MDA-5–MAVS signaling rather than TLR3–TRIF or cGAS–STING signaling, a finding that may suggest more precise therapeutic targets for COVID-19. In addition, we reported that the SARS-CoV-2 M protein inhibits IRF3 phosphorylation and nuclear translocation induced by SeV infection. Last, although the SARS-CoV-1 and MERS-CoV M proteins have been reported to suppress type I IFNs, their roles in viral replication are unknown. We demonstrated that overexpression of the SARS-CoV-2 M protein can promote VSV-eGFP replication.

Although we provide ample results demonstrating that the SARS-CoV-2 M protein inhibits type I and III IFN production, we are aware that ectopic expression of a viral protein differs from that in an in vivo infection in terms of studying its biological function. Since the M protein is a structural protein and indispensable for virion assembly, an M-null SARS-CoV-2 strain is currently unavailable. Identification of the SARS-CoV-2 M protein mutation that results in the loss of IFN suppression abilities but does not affect virion assembly merits further investigation. In addition, this mutant may provide guidelines for future production of M-defective mutant SARS-CoV-2 virions, which would contribute to our understanding of SARS-CoV-2 M in antagonizing IFN production in the context of in vivo viral infection.

Administration of type I or III IFNs alone or in combination with other drugs results in a reduced virus titer, a limited inflammatory response, and mild clinical disease in both animal models and patients infected with SARS-CoV-1 or SARS-CoV-2.^9,28^ Multiple proteins encoded by coronaviruses have been shown to counteract the IFN response, although the detailed action mechanisms of most of these proteins have not been well documented.^8,16,19,38^ An improved understanding of the viral IFN antagonists involved in SARS-CoV-2 pathogenesis has important implications for the development of new antiviral drugs and vaccines. This study was the first to investigate the function of the SARS-CoV-2 M protein in counteracting IFN-mediated innate antiviral immunity and its role in viral replication. Our investigation extends the understanding of strategies employed by SARS-CoV-2 to evade antiviral immunity and provides novel mechanistic insight into the mechanisms by which coronavirus M proteins inhibit IFN production, thus shedding light on the interactions between human antiviral immunity and SARS-CoV-2 infection in the pathogenesis of COVID-19.

## Materials and methods

### Cell culture and transfection

HEK293, HEK293T, HeLa, and Vero cells were cultured in Dulbecco’s modified Eagle’s medium (DMEM, Gibco, USA) supplemented with 10% heat-inactivated fetal bovine serum (FBS, Gibco, USA). All cells were cultured at 37 °C in a humidified incubator containing 5% CO_2_. Plasmids were transfected into HEK293, HEK293T, and HeLa cells with polyethyleneimine ‘Max’ (Polysciences, Inc., Germany). Poly (I:C) (P1530, Sigma-Aldrich, USA) was transfected into cells using Lipofectamine 2000 (Thermo Fisher Scientific, USA) as described previously^33^.

### Antibodies and reagents

Rabbit anti-DYKDDDDK tag (D6W5B), rabbit anti-RIG-I (D14G6), rabbit anti-IRF3 (D83B9), rabbit anti-pIRF3 (4D46), rabbit anti-TBK1 (3031S), rabbit anti-pTBK1 (D52C2), and rabbit anti-TRAF3 antibodies (Abs) were obtained from Cell Signaling Technology (USA); the mouse anti-MAVS (E-3) and mouse anti-TRAF3 (G-6) antibodies were obtained from Santa Cruz Biotechnology (USA); the mouse anti-actin and rabbit anti-calnexin antibodies were obtained from Proteintech (Wuhan, China); the mouse anti-Flag M2 antibody was obtained from Sigma-Aldrich (USA); the mouse anti-Myc (9E10) antibody was obtained from Origene (USA); the rabbit anti-GM130 antibody was obtained from Abcam (United Kingdom); the mouse anti-GAPDH antibody (AF0006) was obtained from Beyotime (China); and the mouse anti-HA antibody was obtained from MDL Biotech (China). Alexa Fluor 488 goat anti-rabbit IgG secondary antibody, Alexa Fluor 568 goat anti-mouse IgG secondary antibody, Alexa Fluor 488 goat anti-mouse IgG secondary antibody, and Alexa Fluor 568 goat anti-rabbit IgG secondary antibody were obtained from Thermo Fisher Scientific. Protein A/G beads were obtained from Santa Cruz Biotechnology, and anti-Flag magnetic beads were obtained from Bimake (USA). Recombinant proteins of human IFN-β (P5660) and human IFN-λ1 (P5669) were purchased from Beyotime (China).

### Constructs and plasmids

The RIG-I, RIG-IN, MDA-5, MAVS, TBK1, IKKε, IRF3-5D, TRIF, and STING sequences were cloned into the pcDNA6B-Flag, pcDNA6B-Myc, pcDNA6B-V5, pCAG-Flag, or pCMV-HA-N expression vectors using standard molecular cloning methods as described in our previous publications.^42–44^ The IFN-β luciferase reporter plasmid pGL3–IFN-β–Luc was constructed in our previous study.^45,46^ The IFN-λ1 luciferase reporter plasmid pGL3–IFN-λ1–Luc was constructed by inserting the 1000-bp promoter region of human IFN-λ1 (nucleotides −1000 to +1, with the translation start site set at nucleotide position 1) into the pGL3-Basic plasmid (Promega, USA) according to a previous study.^13,34^ The ISG luciferase reporter plasmid pISRE-Luc was purchased from Clontech (USA). The SARS-CoV-2 M protein gene sequence (NCBI accession no. MN908947) was synthesized (General Biol, China) and subcloned into the pCAG-Flag expression vector. The sequences of the primers used for plasmid construction are listed in Supplementary Table 1.

### Real-time quantitative PCR

Total RNA isolated with TRIzol Reagent (Invitrogen) was reverse transcribed to first-strand cDNA with a HiScript III 1st Strand cDNA Synthesis Kit with gDNA Wiper (Vazyme, China) according to the manufacturer’s instructions. Real-time quantitative PCR (RT-qPCR) assays were performed using a SYBR Green-based RT-qPCR Kit with UltraSYBR Mixture (CWBIO, China) in a Roche LightCycler 96 system according to the manufacturer’s instructions. The relative abundances of the indicated mRNA transcripts were normalized to that of GAPDH. The comparative C_T_ (ΔΔC_T_) method was used to calculate the fold changes in gene expression levels as described.^44,47^ The primers used in RT-qPCR analysis are listed in Supplemental Table 1.

### Luciferase reporter assays

To assess luciferase reporter activity, including the activity of IFN-β-Luc, IFN-λ1-Luc, and ISRE-Luc, induced by the indicated proteins in each experiment, a dual‐luciferase reporter assay was performed as described in our previous studies.^44,47^ In brief, ~0.5 × 10^5^ HEK293T cells were seeded in 48-well plates. Twelve hours later, cells were transfected with the luciferase reporter plasmid and with the RIG-I, RIG-IN, MDA-5, MAVS, TBK1, IKKε, IRF3-5D, TRIF, and STING expression plasmids alone or together with the SARS-CoV-2 M protein expression plasmid, as indicated in the experiments. The pRL-TK Renilla luciferase reporter plasmid (Promega, USA) was cotransfected for normalization of the transfection efficiency and was the internal control. Thirty-six hours after transfection, cells were harvested and lysed for assessment of luciferase activity with a Dual Luciferase Reporter Assay Kit (Vazyme, China) according to the manufacturer’s protocol. Luciferase activity was measured in a Centro XS3 LB 960 microplate luminometer (Berthold Technologies, Germany). Relative luciferase activity was calculated by normalizing firefly luciferase activity to Renilla luciferase activity.

### Viruses and infection

Enhanced green fluorescent protein (eGFP)-labeled VSV and SeV were used to infect HeLa, HEK293, or HEK293T cells as described in our previous publications.^42–44^ In brief, before infection, target cells were washed with serum‐free DMEM prewarmed to 37 °C, and the virus was then diluted to the desired MOI in serum‐free DMEM and incubated with the target cells for 1–2 h. At the end of the infection step, the virus‐medium complexes were discarded, and DMEM containing 10% FBS was added.

### Co-IP and immunoblotting

For the Co-IP assay, HEK293T cells were collected 24 h after transfection and lysed in lysis buffer [1.0% (v/v) NP-40, 50 mM Tris–HCl (pH 7.4), 50 mM EDTA, 0.15 M NaCl] supplemented with a protease inhibitor cocktail (Sigma-Aldrich) and a phosphatase inhibitor cocktail (Sigma-Aldrich) as described in our previous publications.^42,43^ After centrifugation for 10 min at 14,000 × *g*, supernatants were collected and incubated with the indicated antibodies. Protein A/G beads (Santa Cruz), anti-Flag magnetic beads (Bimake) or anti-Myc magnetic beads (Bimake) were then added. After incubation overnight at 4 °C, the beads were washed four times with lysis buffer. Immunoprecipitates were eluted by boiling with 2 × SDS loading buffer containing 100 mM Tris–HCl (pH 6.8), 4% (w/v) SDS, 20% (v/v) glycerol, 0.2% (w/v) bromophenol blue, and 1% (v/v) 2-mercaptoethanol.

For immunoblot analysis, the M-PER Protein Extraction Reagent (Pierce, USA) supplemented with a protease inhibitor cocktail (Sigma, USA) was used to lyse the cells. The protein concentrations in the lysates were measured with a bicinchoninic acid assay (Pierce, USA), and the samples were brought to equal concentrations with extraction reagent. Total cell lysates or immunoprecipitates prepared as described above were electrophoretically separated by SDS–PAGE, transferred to a polyvinylidene difluoride membrane (Millipore, Germany), blocked with 3% (w/v) bovine serum albumin (BSA), reacted with the indicated primary antibodies and corresponding secondary antibodies, and visualized with ECL Western blotting detection reagent (Pierce, USA).

### Confocal immunofluorescence microscopy

Confocal immunofluorescence microscopy was performed as described in our previous publications.^42,43^ In briefly, HeLa cells were grown on 12-well slides one day before transfection with the indicated plasmids. Transfected or infected HeLa cells were then fixed with 4% paraformaldehyde, permeabilized with 0.2% Triton X-100, and blocked with phosphate-buffered saline (PBS) containing 5% horse serum and 1% BSA. The fixation, permeabilization, and blocking buffers were purchased from Beyotime Biotechnology (China). The cells were then incubated with the indicated primary antibodies at 4 °C overnight, rinsed, and incubated with the corresponding secondary antibodies (Invitrogen, USA). Nuclei were counterstained with DAPI (Abcam). Images were acquired with a Zeiss LSM780 confocal microscope (Germany).

### Viral plaque assays

Viral plaque assays were performed on Vero-E6 cells to measure the titer of VSV-eGFP as described in our previous study.^44^ In brief, Vero cells were seeded on 24-well plates. The next day, cells at a confluence of ~100% were infected with serial dilutions of VSV-eGFP for 30 min. After infection, the medium was replaced with DMEM containing 0.5% agar and 2% FBS. After the agar overlay solidified, cells were cultured for 20–24 h and were then fixed with a 1:1 methanol–ethanol mixture for 30 min. After the solid agarose-medium mix was removed, the cells were stained with 0.05% crystal violet, and the plaques on the monolayer were then counted to calculate the viral titer.

### Flow cytometry analysis

After infections, cells were harvested by trypsination and resuspended in PBS with 0.5% BSA and 2 mM EDTA buffer. The cells were gated for GFP signals based on the background signal from the non-infected cells. Fluorescent intensity was determined on a Beckman Coulter Gallios flow cytometer with at least 10,000 cells per sample. Data analysis was carried out with FlowJo software.

### Bioinformatic analysis

The transmembrane motifs were predicted with TMHMM server version 2.0 (http://www.cbs.dtu.dk/services/TMHMM/).

### Statistical analysis

The results are representative of three independent experiments and are presented as the mean ± SD values. For statistical analysis, two‐tailed unpaired Student’s *t*‐tests were performed in GraphPad Prism 8.0 and Microsoft Excel. *P* values are shown in each figure or figure legend. In all cases, a value of *P* < 0.05 was considered to indicate a statistically significant difference.

## Supplementary information

Supplementary Materials

## Data Availability

All data and materials are available to the researchers once published.

## References

[1] Wu F (2020). A new coronavirus associated with human respiratory disease in China. Nature.

[2] Zhou P (2020). A pneumonia outbreak associated with a new coronavirus of probable bat origin. Nature.

[3] Zhu N (2020). A novel coronavirus from patients with pneumonia in China, 2019. N. Engl. J. Med..

[4] Cui J, Li F, Shi ZL (2019). Origin and evolution of pathogenic coronaviruses. Nat. Rev. Microbiol..

[5] Kim D (2020). The architecture of SARS-CoV-2 transcriptome. Cell.

[6] Blanco-Melo D (2020). Imbalanced host response to SARS-CoV-2 drives development of COVID-19. Cell.

[7] Ni L (2020). Detection of SARS-CoV-2-specific humoral and cellular immunity in COVID-19 convalescent individuals. Immunity.

[8] Totura AL, Baric RS (2012). SARS coronavirus pathogenesis: host innate immune responses and viral antagonism of interferon. Curr. Opin. Virol..

[9] Park A, Iwasaki A (2020). Type I and Type III Interferons—induction, signaling, evasion, and application to combat COVID-19. Cell Host Microbe.

[10] Hu YH (2015). WDFY1 mediates TLR3/4 signaling by recruiting TRIF. EMBO Rep..

[11] Liu S (2015). Phosphorylation of innate immune adaptor proteins MAVS, STING, and TRIF induces IRF3 activation. Science.

[12] Sun L (2013). Cyclic GMP-AMP synthase is a cytosolic DNA sensor that activates the type I interferon pathway. Science.

[13] Onoguchi K (2007). Viral infections activate types I and III interferon genes through a common mechanism. J. Biol. Chem..

[14] Chang CY, Liu HM, Chang MF, Chang SC (2020). Middle East respiratory syndrome coronavirus nucleocapsid protein suppresses Type I and Type III interferon induction by targeting RIG-I signaling. J. Virol..

[15] Yoshikawa T (2010). Dynamic innate immune responses of human bronchial epithelial cells to severe acute respiratory syndrome-associated coronavirus infection. PLoS ONE.

[16] Lei X (2020). Activation and evasion of type I interferon responses by SARS-CoV-2. Nat. Commun..

[17] Hadjadj J (2020). Impaired type I interferon activity and inflammatory responses in severe COVID-19 patients. Science.

[18] Zhou Z (2020). Heightened innate immune responses in the respiratory tract of COVID-19 patients. Cell Host Microbe.

[19] Broggi A (2020). Type III interferons disrupt the lung epithelial barrier upon viral recognition. Science.

[20] Lucas C (2020). Longitudinal analyses reveal immunological misfiring in severe COVID-19. Nature.

[21] Hung IF (2020). Triple combination of interferon beta-1b, lopinavir-ritonavir, and ribavirin in the treatment of patients admitted to hospital with COVID-19: an open-label, randomised, phase 2 trial. Lancet.

[22] Irvani SSN (2020). Effectiveness of Interferon Beta 1a, compared to Interferon Beta 1b and the usual therapeutic regimen to treat adults with moderate to severe COVID-19: structured summary of a study protocol for a randomized controlled trial. Trials.

[23] Mantlo E (2020). Antiviral activities of type I interferons to SARS-CoV-2 infection. Antivir. Res..

[24] Zhongji Meng, T. W. et al. An experimental trial of recombinant human interferon alpha nasal drops to prevent coronavirus disease 2019 in medical staff in an epidemic area. Preprint at 10.1101/2020.04.11.20061473 (2020).

[25] Lokugamage KG (2020). Type I Interferon Susceptibility Distinguishes SARS-CoV-2 from SARS-CoV. J. Virol..

[26] Vanderheiden A (2020). Type I and Type III Interferons Restrict SARS-CoV-2 Infection of Human Airway Epithelial Cultures. J. Virol..

[27] O’Brien TR (2020). Weak induction of interferon expression by SARS-CoV-2 supports clinical trials of interferon lambda to treat early COVID-19. Clin. Infect. Dis..

[28] Stanifer ML (2020). Critical role of Type III interferon in controlling SARS-CoV-2 infection in human intestinal epithelial cells. Cell Rep..

[29] Davidson S (2016). IFNlambda is a potent anti-influenza therapeutic without the inflammatory side effects of IFNalpha treatment. EMBO Mol. Med..

[30] Lazear HM, Schoggins JW, Diamond MS (2019). Shared and distinct functions of Type I and Type III Interferons. Immunity.

[31] Fu Hsin, et al. View ORCID ProfileHelene Minyi Liu. Distinct inductions of and responses to Type I and Type III interferons promote infections in two SARS-CoV-2 isolates. Preprint at 10.1101/2020.04.30.071357 (2020).

[32] Ziegler CGK (2020). SARS-CoV-2 receptor ACE2 is an interferon-stimulated gene in human airway epithelial cells and is detected in specific cell subsets across tissues. Cell.

[33] Zhuang MW (2020). Increasing host cellular receptor-angiotensin-converting enzyme 2 expression by coronavirus may facilitate 2019-nCoV (or SARS-CoV-2) infection. J. Med. Virol..

[34] Siu KL (2009). Severe acute respiratory syndrome coronavirus M protein inhibits type I interferon production by impeding the formation of TRAF3.TANK.TBK1/IKKepsilon complex. J. Biol. Chem..

[35] Lui PY (2016). Middle East respiratory syndrome coronavirus M protein suppresses type I interferon expression through the inhibition of TBK1-dependent phosphorylation of IRF3. Emerg. Microbes Infect..

[36] Wang B (2020). Long-term coexistence of SARS-CoV-2 with antibody response in COVID-19 patients. J. Med. Virol..

[37] Siu KL (2014). Suppression of innate antiviral response by severe acute respiratory syndrome coronavirus M protein is mediated through the first transmembrane domain. Cell Mol. Immunol..

[38] Yuen CK (2020). SARS-CoV-2 nsp13, nsp14, nsp15 and orf6 function as potent interferon antagonists. Emerg. Microbes Infect..

[39] Han, L. et al. SARS-CoV-2 ORF9b antagonizes Type I and III interferons by targeting multiple components of RIG-I/MDA-5-MAVS, TLR3-TRIF, and cGAS-STING signaling pathways. Preprint at 10.1101/2020.08.16.252973 (2020). 10.1002/jmv.27050 PMC8242602 33913550

[40] Tao Y, Yang Y, Zhou R, Gong T (2020). Golgi apparatus: an emerging platform for innate immunity. Trends Cell Biol..

[41] Pourcelot M (2016). The Golgi apparatus acts as a platform for TBK1 activation after viral RNA sensing. BMC Biol..

[42] Liu B (2017). The ubiquitin E3 ligase TRIM31 promotes aggregation and activation of the signaling adaptor MAVS through Lys63-linked polyubiquitination. Nat. Immunol..

[43] Song G (2016). E3 ubiquitin ligase RNF128 promotes innate antiviral immunity through K63-linked ubiquitination of TBK1. Nat. Immunol..

[44] Wang PH (2018). A novel transcript isoform of STING that sequesters cGAMP and dominantly inhibits innate nucleic acid sensing. Nucleic Acids Res..

[45] Wang PH (2013). Nucleic acid-induced antiviral immunity in shrimp. Antivir. Res..

[46] Wang PH (2013). The shrimp IKK-NF-kappaB signaling pathway regulates antimicrobial peptide expression and may be subverted by white spot syndrome virus to facilitate viral gene expression. Cell Mol. Immunol..

[47] Wang PH (2018). Inhibition of AIM2 inflammasome activation by a novel transcript isoform of IFI16. EMBO Rep..

