# Supplementary material for: Severe acute respiratory syndrome coronavirus 2 (SARS-CoV-2) membrane (M) protein inhibits type I and III interferon production by targeting RIG-I/MDA-5 signaling
Source: Signal Transduct Target Ther. 2020 Dec 28;5:299. doi: 10.1038/s41392-020-00438-7 (PMC7768267; doi:10.1038/s41392-020-00438-7)
Supplement: Supplementary file 1 — Supplementary Materials [file 41392_2020_438_MOESM1_ESM.docx]

Supplementary Materials for

Severe Acute Respiratory Syndrome Coronavirus 2 (SARS-CoV-2) Membrane (M) Protein Inhibits Type I and III Interferon Production by Targeting RIG-I/MDA-5 Signaling

Yi Zheng ^1^, Meng-Wei Zhuang ^2^, Lulu Han ^1^, Jing Zhang ^2^, Mei-Ling Nan ^2^, Peng, Zhan ^3, 4^, Dongwei Kang ^3, 4^, Xinyong Liu ^3, 4^, Chengjiang Gao ^1^, Pei-Hui Wang ^2, 5^

^1^ Key Laboratory of Infection and Immunity of Shandong Province, Department of Immunology, School of Basic Medical Sciences, Cheeloo College of Medicine, Shandong University, Jinan 250012, China

^2^ Key Laboratory for Experimental Teratology of Ministry of Education and Advanced Medical Research Institute, Cheeloo College of Medicine, Shandong University, Jinan 250012, China

^3^ Department of Medicinal Chemistry, Key Laboratory of Chemical Biology (Ministry of Education), School of Pharmaceutical Sciences, Cheeloo College of Medicine, Shandong University, 44 West Culture Road, 250012 Jinan, Shandong, PR China

^4^ China-Belgium Collaborative Research Center for Innovative Antiviral Drugs of Shandong Province, 44 West Culture Road, 250012 Jinan, Shandong, PR China

^5^ Suzhou Research Institute, Shandong University, Shandong University, Suzhou, Jiangsu 215123, China

These authors contributed equally: Yi Zheng, Meng-Wei Zhuang

Correspondence to: Chengjiang Gao ([cgao@sdu.edu.cn](mailto:cgao@sdu.edu.cn)) or Pei-Hui Wang ([pei-hui.wang@sdu.edu.cn](mailto:pei-hui.wang@sdu.edu.cn) or [pei-hui.wang@connect.hku.hk](mailto:pei-hui.wang@connect.hku.hk))

**This PDF file includes:**

Figures. S1 to S4

Tables S1


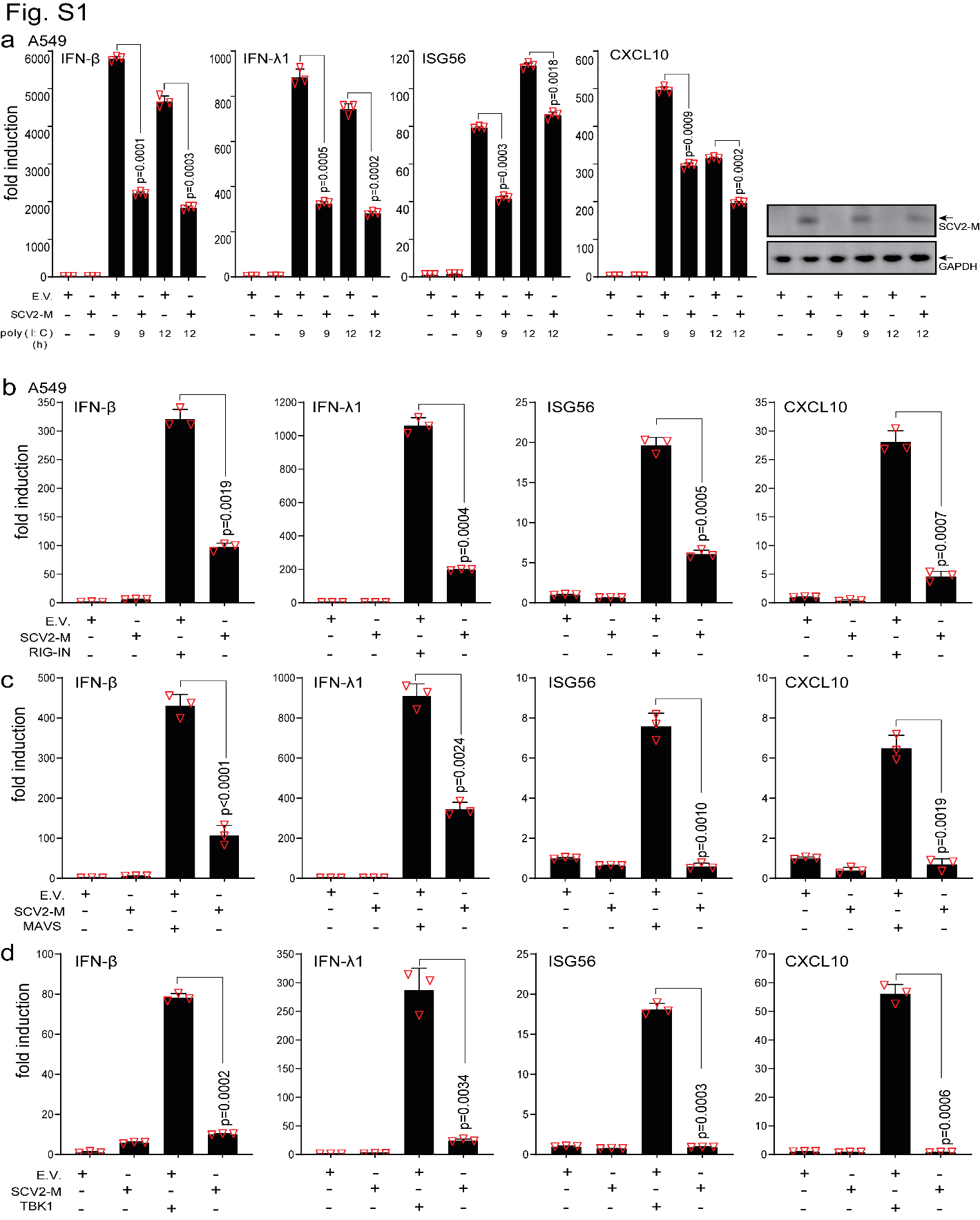


Figure. S1.

**The SARS-CoV-2 M protein inhibits the induction of type I and III IFNs in A549 cells. a.** A549 cells cultured in 24-well plates (0.8-1 × 10^5^ cells per well) were transfected with the empty vector pcDNA6B (E.V., 500 ng) or SARS-CoV-2 M protein-expressing plasmid (500 ng). Twenty-four hours after transfection, cells were stimulated by poly (I:C) (1000 ng/mL) transfection as indicated; 9 and 12 hours after stimulation, cells were harvested for RNA extraction and subsequent RT-qPCR analysis. **b-d.** A549 cells were transfected with plasmids as indicated, 36 hours later, cells were harvested for RT-qPCR analysis. Three independent biological replicates were analyzed, the results of one representative experiment are shown, and the error bars indicate the SD values. The statistical significance is shown as indicated. SARS-CoV-2 M protein, SCV2-M; hours, h.


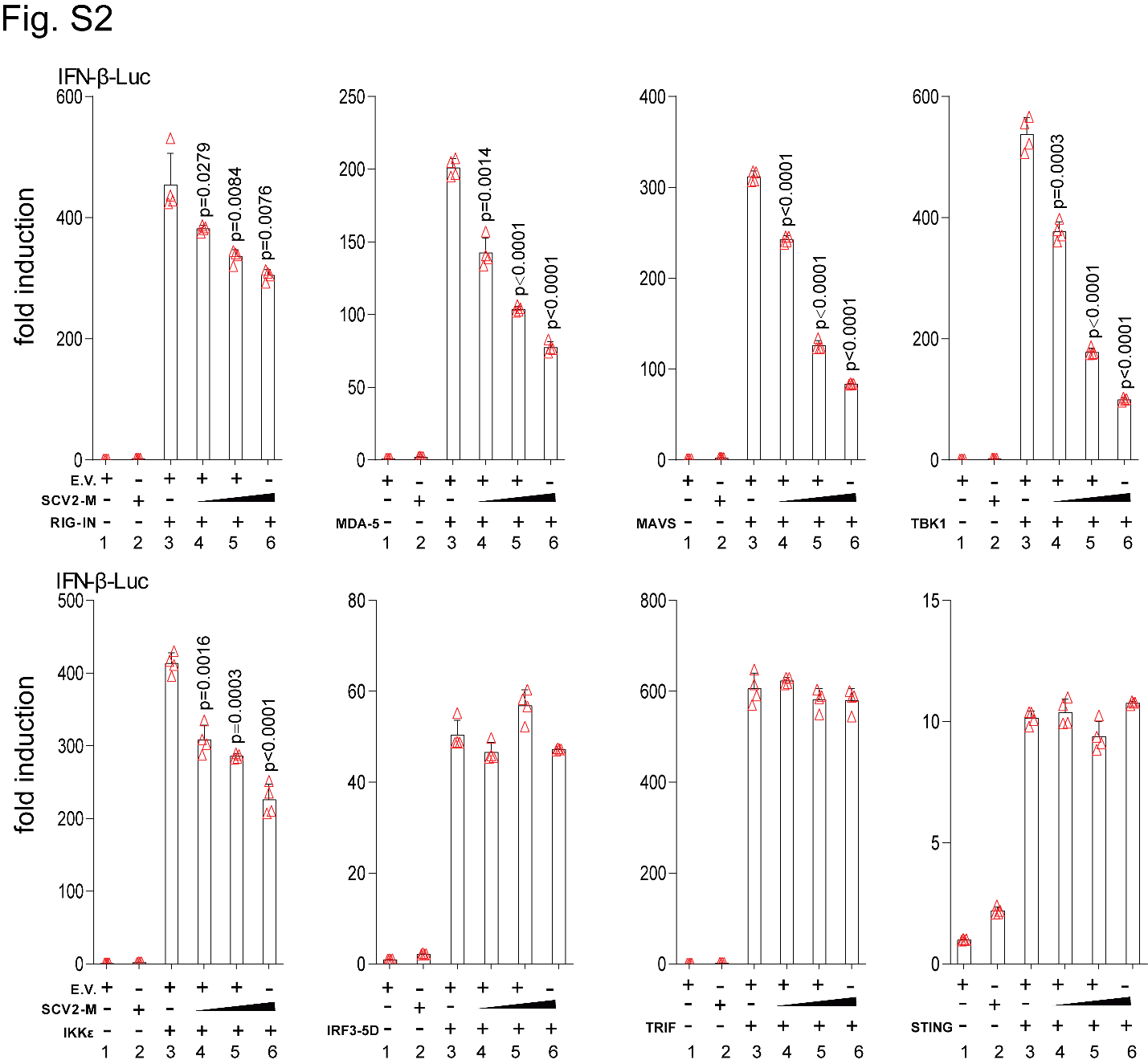


Figure. S2.

**The SARS-CoV-2 M protein inhibits the activation of IFN-β-Luc by** **RIG-IN, MDA-5, MAVS, TBK1, and IKKε in a dose-dependent manner.** Plasmids of IFN-β-Luc (23 ng) and pRL-TK (2 ng) were transiently transfected with plasmids expressing RIG-IN (50 ng), MDA-5 (50 ng), MAVS (50 ng), TBK1 (50 ng), IKKε (50 ng), IRF3-5D (50 ng), TRIF (50 ng), or STING (50 ng) into HEK293T cells cultured in 48-well plates as indicated. Increasing doses of SCV2-M expressing plasmid (50 ng in lane 4, 100 ng in lane 5, 125 ng in lanes 2 and 6,) were co‐transfected. Empty vector pcDNA6B-Flag was used to balance total amount of transfected DNA. Cell lysates were assessed 36 hrs after transfection for luciferase activity. Three independent biological replicates were analyzed; the results of one representative experiment are shown, and the error bars indicate SD. The statistical significance is shown as indicated. Empty vector, E.V.; SARS-CoV-2 M protein, SCV2-M.


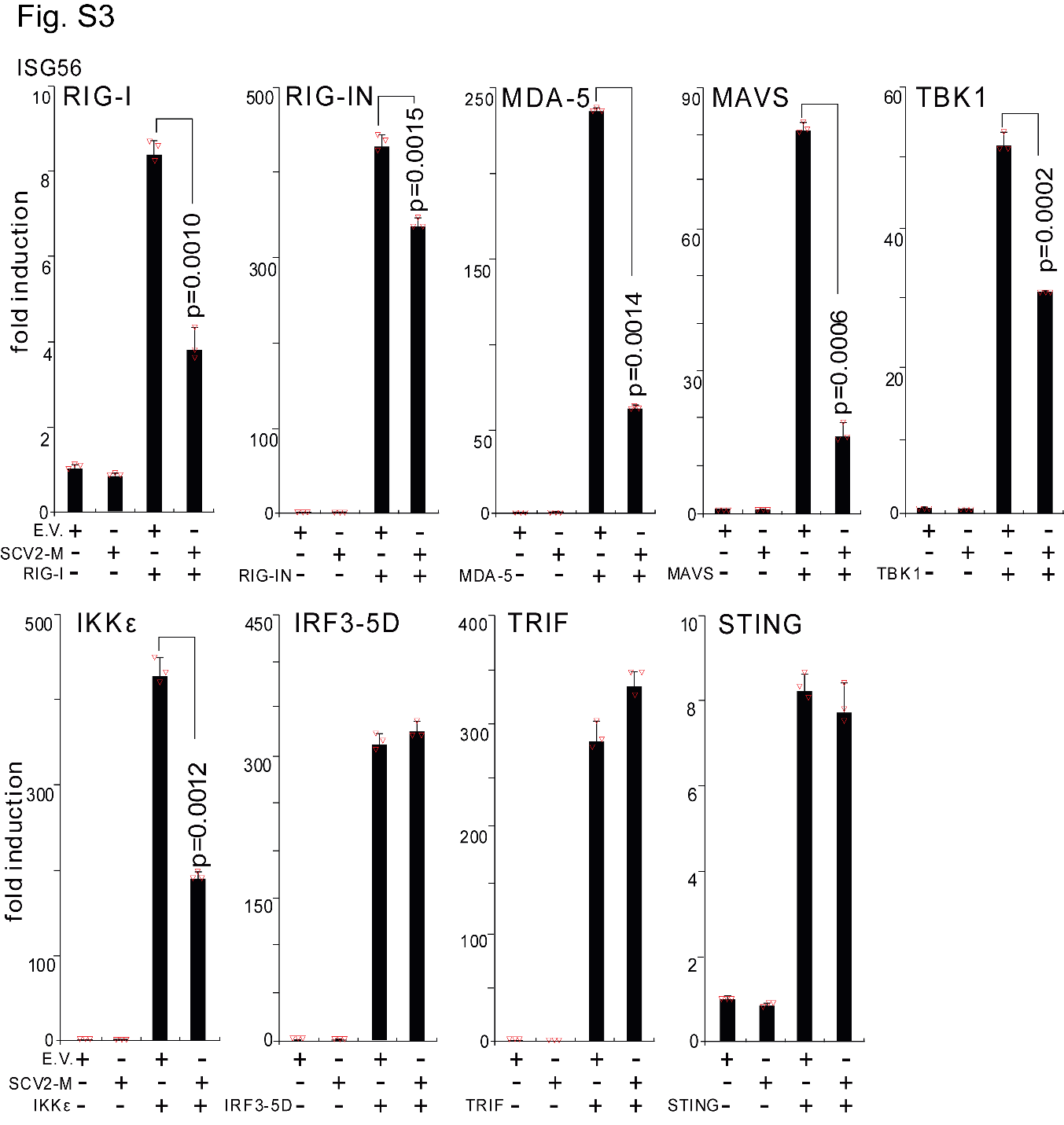


Figure. S3.

**The SARS-CoV-2 M protein impairs ISG56 production induced by the RIG-I/MDA-5 pathway but not by the TLR3-TRIF or cGAS-STING pathways.** The pcDNA6B empty vector and SARS-CoV-2 M plasmid (300 ng) were transfected with the indicated combinations of plasmids expressing RIG-IN (200 ng), MDA-5 (200 ng), TBK1 (200 ng), IKKε (200 ng), IRF3-5D (200 ng), TRIF (200 ng), or STING (200 ng) into HEK293T cells cultured in 24-well plates (0.8-1 × 10^5^ cells per well). Empty vector pcDNA6B-Flag was used to balance total amount of transfected DNA. Thirty-six hours later, cells were harvested for RNA extraction and subsequent RT-qPCR analysis of the ISG56 induction. Three independent biological replicates were analyzed; the results of one representative experiment are shown, and the error bars indicate SD. The statistical significance is shown as indicated. Empty vector, E.V.; SARS-CoV-2 M protein, SCV2-M.


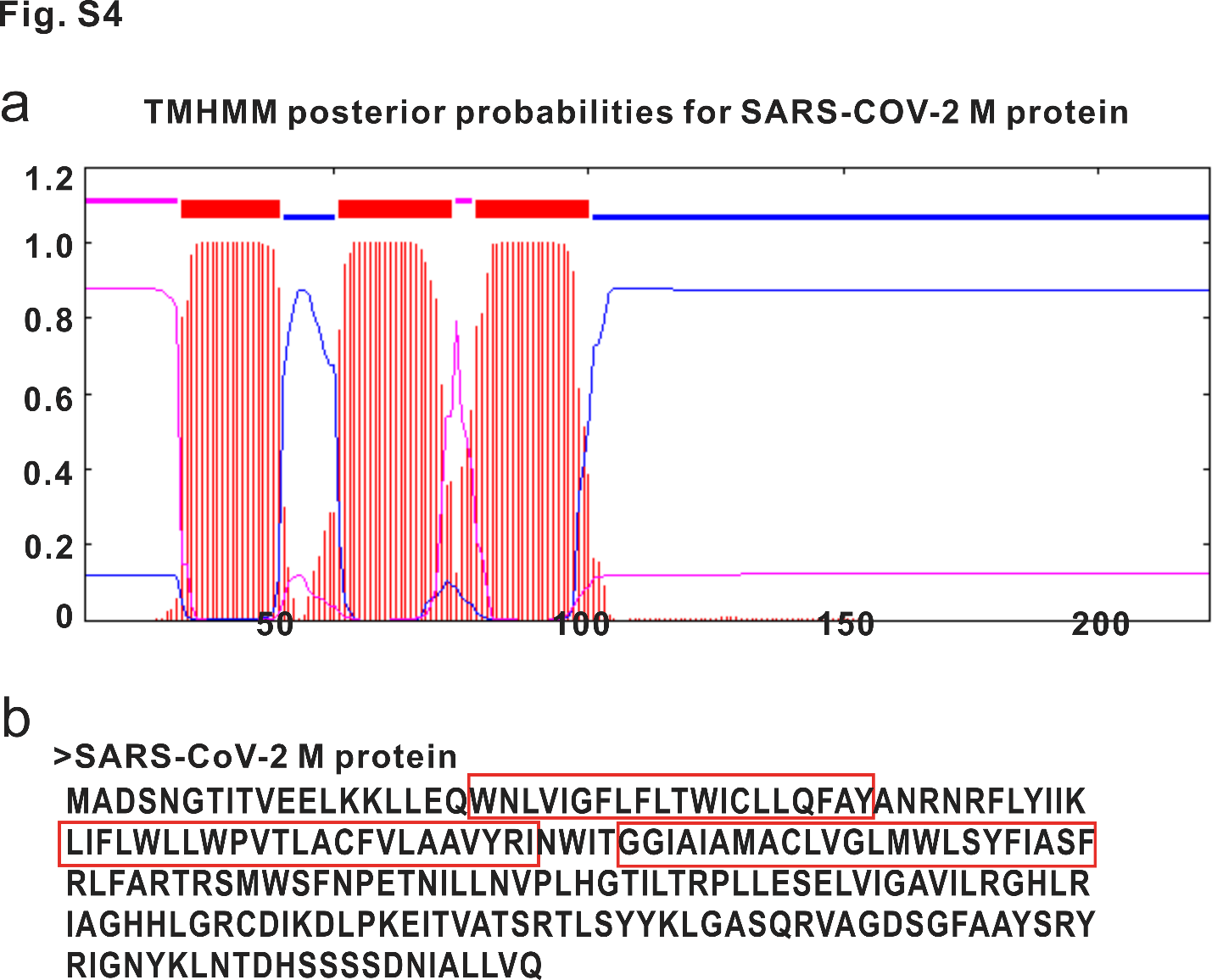


Figure. S4.

**The SARS-CoV-2 M protein is predicted to contain three transmembrane motifs.** **a.** The transmembrane motifs in the SARS-CoV-2 M protein were predicted with the TMHMM server, version 2.0. **b.** The transmembrane motifs in the SARS-CoV-2 M protein are enclosed in red boxes.

Table S1.

Primers used in this study.

| Primer name | Sequence (5′-3′) | Usage |
| --- | --- | --- |
| GAPDH-F  GAPDH-R | GGAGCGAGATCCCTCCAAAAT  GGCTGTTGTCATACTTCTCATGG | RT-qPCR |
| IFN-β-F  IFN-β-R | TTGCTCTCCTGTTGTGCTTC  AAGCCTCCCATTCAATTGCC | RT-qPCR |
| IFN-λ1-F  IFN-λ1-R | GAGGCCCCCAAAAAGGAGTC  AGGTTCCCATCGGCCACATA | RT-qPCR |
| ISG56-F  ISG56-R | CTAAGCAAAACCCTGCAGAAC  TCAGGCATTTCATCGTCATC | RT-qPCR |
| CXCL10-F  CXCL10-R | GTGGCATTCAAGGAGTACCTC  GACCTTTCCTTGCTAACTGCT | RT-qPCR |
| SCV2-M-F  SCV2-M-R | GCACAGTGGCGGCCGCTCGAGGCCACCATGGCAGATTCCAACGGTAC  GTCATCCTTGTAATCTCTAGACTGTACAAGCAAAGCAATATTG | expression plasmid |
| IFN-λ1-Luc-F  IFN-λ1-Luc-R | GGGGTACCTAAACCAATGGCAGAAGCTCC  GAAGATCTGGCTAAATCGCAACTGCTTCCCCAG | luciferase reporter |

F: forward primer. R: reverse primer.
